# Development of mini-SSPedi for children 4–7 years of age receiving cancer treatments

**DOI:** 10.1186/s12885-018-5210-z

**Published:** 2019-01-08

**Authors:** Deborah Tomlinson, Shannon Hyslop, Eliana Stein, Brenda Spiegler, Emily Vettese, Susan Kuczynski, Tal Schechter, L. Lee Dupuis, Lillian Sung

**Affiliations:** 1 Child Health Evaluative Sciences, The Hospital for Sick Children, Peter Gilgan Centre for Research and Learning, Toronto, Ontario M5G 0A4 Canada; 2 Department of Psychology, The Hospital for Sick Children, Toronto, Canada; 3 Ontario Parents Advocating for Children with Cancer (OPACC), Toronto, Canada; 4 Department of Pharmacy, The Hospital for Sick Children, Toronto, Canada; 5 Leslie Dan Faculty of Pharmacy, University of Toronto, Toronto, Canada; 6 Division of Haematology and Oncology, The Hospital for Sick Children, Toronto, Canada

**Keywords:** Symptom screening, Children, Self-report, Cancer, Hematopoietic stem cell transplantation

## Abstract

**Background:**

The Symptom Screening in Pediatrics Tool (SSPedi) is valid for assessing symptoms in children aged 8–18 years receiving cancer treatments. The objective was to develop a new self-report symptom screening tool for children receiving cancer treatments who are 4–7 years of age (mini-SSPedi), based on SSPedi.

**Methods:**

Respondents were children with cancer or pediatric hematopoietic stem cell transplantation (HSCT) recipients who were 4–7 years of age. We included the same 15 symptoms contained in SSPedi. Using cognitive interviewing, we developed mini-SSPedi in three phases and made decisions based upon respondent understanding. First, we developed questionnaire structure regarding recall period, concept of bother and response option format. Second, we determined wording of each symptom. Third, we evaluated the entire mini-SSPedi instrument for understanding and ease of completion.

**Results:**

We enrolled 100 participants in total and included 30, 40 and 30 in each of the three phases. Questionnaire structure was satisfactory with a recall period of “today” and a faces-based 3-point Likert scale. Bother was well-understood. Five symptoms required modification to achieve satisfactory understanding while the remaining 10 SSPedi symptoms did not require modification. Among the last 10 children enrolled, all understood each mini-SSPedi item and none thought mini-SSPedi was hard to complete.

**Conclusion:**

We developed a symptom screening tool for children with cancer and pediatric HSCT recipients between 4 and 7 years of age that is understandable and easy to complete. Future work will evaluate the psychometric properties of mini-SSPedi and develop an electronic version of the instrument.

**Electronic supplementary material:**

The online version of this article (10.1186/s12885-018-5210-z) contains supplementary material, which is available to authorized users.

## Background

Over the last few decades, impressive gains in survival for children and adolescents with cancer have been made and now, more than 82% of children with cancer will be cured [1]. These survival gains have been, in part, attributable to the provision of intensive therapies. However, as a result, most children suffer and experience severe and distressing treatment-related symptoms such as pain, fatigue and nausea [2]. In our recent cross-sectional study of 302 inpatients 8–18 years of age, when asked about yesterday or today, 99% of children experienced at least one bothersome symptom and 60% experienced at least one *severely* bothersome symptom, including severe pain in 22% and severe fatigue in 33% [3]. Given excellent survival outcomes, we now need to focus more attention on symptom control. Active symptom screening and ongoing assessment are likely to be important components of successful symptom control in children with cancer. We previously identified the need for a new self-report symptom screening tool for children receiving cancer treatments [4] and thus developed the Symptom Screening in Pediatrics Tool (SSPedi) for children 8 to 18 years of age [4, 5]. SSPedi is reliable, valid and responsive to change in this age group [3].

However, SSPedi does not address the needs of children younger than 8 years of age. A reliable and valid patient-reported outcome scale for younger children receiving cancer treatments is lacking [6]. Without such a tool, we will not know how symptoms related to diagnosis or treatment differ among younger children. Consequently, there is a need to develop or modify a symptom screening tool for this patient population. Developing patient-reported outcomes for young children is expected to be more challenging than for older cohorts. Representation abilities develop at around age 3–5 years, with the ability of introspection about one’s own thoughts developing at age 6–8 years [7, 8]. Since children as young as 4 or 5 years of age are thought to be capable of describing concrete aspects about their health [7], our efforts began with children who were 4 years of age. We based this instrument on SSPedi to allow comparability between age groups and named our instrument for children receiving cancer treatments who were 4–7 years of age mini-SSPedi.

The objective was to develop a new self-report symptom screening tool for children receiving cancer treatments who are 4–7 years of age (mini-SSPedi), based on SSPedi.

## Methods

### Overall approach

The study applied an exploratory design. The clinical research nurse (DT) conducted qualitative cognitive interviews with child participants, using methodology previously used in young children [8–11]. The interview started with clarification on our purpose, namely to teach us the best way to ask children about how they feel [9]. We used both closed and open ended questions. Initially, we engaged the child with direct easy-to-answer questions. Then, open-ended questions followed [9].

### Subjects

Eligibility criteria were children 4–7 years of age with cancer or pediatric hematopoietic stem cell transplantation (HSCT) recipients who could understand English. Exclusion criteria were illness severity, cognitive disability or other impairment that precluded completion of mini-SSPedi according to their primary healthcare team. Sampling was purposive to consider variance by age and gender with enrichment of younger children.

### Procedures

Children were recruited from The Hospital for Sick Children (SickKids) in Toronto, Canada. The study received Research Ethics Board approval from SickKids, and parents provided informed consent and children provided assent to participate. Potential respondents were approached by a member of the study team. Demographic information were obtained from parents and from the patient’s health records. Interviews were conducted by a clinical research nurse with experience in cognitive interviewing (DT), while a second team member rated understanding and recorded non-verbal actions. We previously described our approach to cognitive interviewing [5]. For each element, we rated understanding on a 4-point Likert scale ranging from 1 = “completely incorrect” to 4 = “completely correct”. We categorized adequate understanding if a child was mainly or completely correct (score of 3 or 4). All interviews included the use of an ungendered, hand puppet to engage with respondents [12]. We requested that parents be present for the interview but asked them not to intervene and not to help the child. All interviews were audio-recorded and transcribed.

Development of mini-SSPedi was divided into three phases: (1) Establishing questionnaire structure regarding recall period, understanding of “bother” and response scale; (2) Understanding individual symptoms when administered individually; and (3) Understanding of mini-SSPedi when administered in its entirety. After every five children, the research team met to review findings and to determine whether the script or minor edits to mini-SSPedi were required. After every 10 to 20 participants, a Review Panel composed of a pediatric oncology nurse (DT), clinical psychologist (BS), patient advocate (SK), pediatric oncology pharmacist (LLD) and pediatric oncologist (LS) met to make decisions about mini-SSPedi modifications and to confirm when a phase was satisfactorily completed. A phase was considered complete if at least 80% of respondents were correct in their interpretation of a feature within the last group of 10 children interviewed and if comments did not suggest that changes were required. Details of the phases are described below.

#### Establishing questionnaire structure

The first step was to establish the structure of mini-SSPedi. To do so, we focused on the recall period (yesterday or today in SSPedi), understanding of the concept of “bother” and the response scale (5-point Likert scale in SSPedi). In order to adjudicate understanding, we focused on the symptom of pain in this phase as children as young as 4 years of age can self-report pain intensity [13, 14]. We provided the following opening statement: “Please think about how much pain bothered you yesterday or today”. We determined the child’s understanding of the time frame described by asking what he or she did or what happened yesterday, and then what happened today. The parent was asked for agreement on his or her response. We specifically asked about the difference between yesterday and today.

Next, we asked children what “bother” meant to them. If they had trouble with this concept, we asked them for examples of things that might bother them and how they might feel when they are bothered. We asked them for other words that mean the same thing as bother.

Finally, we determined response options that were understood by the respondents. SSPedi has a 5-point Likert scale for degree of bother that ranges from 0 = “not at all bothered” to 4 = “extremely bothered”. We started by asking respondents, again using the pain symptom, whether they were bothered by pain using the recall period immediately just tested (initially yesterday or today). This first question had a dichotomous response scale (yes or no). We then asked why the child picked the chosen option to test understanding. If the child did not understand the dichotomous response scale or recall period, we then stopped. If the child understood the dichotomous response scale, we then tested three different 3-point Likert scales consisting of “not at all bothered”, “medium” and “extremely bothered” as we wanted to maintain the same anchors as SSPedi if possible. The scales tested were all pictorial and based on the Wong-Baker FACES pain scale [15], Faces Pain Scale–Revised (FPS-R) [16], and Pieces of Hurt (Poker Chip tool) [15] (Additional file 1). We asked children to indicate how much pain bothered them using the same recall period just tested, and presented the three scales in random order. Finally, we asked their preference between the three different pictorial options.

#### Understanding individual symptoms

After we had established the recall period, understanding of the concept of bother and the response option format, we then tested understanding of the wording of each symptom. As we wanted to maintain symmetry with SSPedi, we used the 15 symptoms present in SSPedi.

To present each symptom, we used a board with a windowed frame that displayed only the symptom being evaluated. The child was given the opportunity to read the symptom himself or herself (with or without assistance) or to ask the interviewer to read the symptom aloud. With each symptom, we performed cognitive probing to determine the child’s understanding of the symptom using the same methodology we used in the development of SSPedi [5]. We allowed the child to ask for synonyms to clarify symptoms; the synonym list evolved with continued cognitive interviews as synonyms used by participants were added. Several of the SSPedi items consisted of two concepts such as disappointed and sad. We adjudicated the symptom as understood if at least one of the two concepts was correctly understood as long as the second concept was not incorrectly interpreted. In the event of poor understanding of two-concept symptoms, the Review Panel adjudicated whether these should be reduced to one item or changed altogether based upon the qualitative responses.

#### Understanding mini-SSPedi

After establishing questionnaire structure and understandable wording of each symptom item, we then presented mini-SSPedi in its entirety. Again, respondents were given the option of completing it on their own or asking the interviewer to read the instrument aloud. When read aloud, the degree of bother for each symptom was read one at a time, with the instrument stem (how much did *symptom* bother you *recall period*) and redirection to the three faces response scale with each symptom.

Once the instrument was well-understood, a final cohort of 10 children were asked to complete mini-SSPedi in this manner, and were asked to judge instrument length and ease of completion. Mini-SSPedi length was rated using a 3-point Likert scale consisting of “too short”, “just right” and “too long”. We also asked if overall, mini-SSPedi was easy or hard to complete also using a 3-point Likert scale anchored by “easy” and “hard”. A parent also rated the child’s ease of completion on a 5-point Likert scale anchored by “very easy” and “very hard”.

## Results

There were 135 children evaluated for participation in this study of which 28 were not eligible and 7 declined to participate. One hundred children were enrolled in nine distinct cohorts between March 2017 and January 2018. There were 30 children enrolled to establish questionnaire structure, 40 children enrolled to establish understanding of each symptom and 30 children enrolled to test understanding of the entire mini-SSPedi instrument. Table 1 illustrates the demographics of the children presented by phase and cohort. Over all cohorts, the mean age (range) was 5.6 (4.0 to 7.8) years and 61 (61.0%) were boys. Median interview time was 14.0 (range 8.3 to 29.0) minutes.

**Table 1:** Demographics of the Study Cohorts (*N* = 100)^a^

	Establishing Questionnaire Structure (*N* = 30)			Understanding Individual Symptoms (*N* = 40)				Understanding Mini-SSPedi (*N* = 30)	
	Cohort 1 (*n* = 10)	Cohort 2 (*n* = 10)	Cohort 3 (*n* = 10)	Cohort 4 (*n* = 10)	Cohort 5 (*n* = 10)	Cohort 6 (*n* = 10)	Cohort 7 (*n* = 10)	Cohort 8 (*n* = 20)	Cohort 9 (*n* = 10)
Age in years									
4	3	5	5	6	1	1	2	7	2
5	3	1	1	3	6	5	5	5	4
6	4	3	3	1	3	1	0	4	3
7	0	1	1	0	0	3	3	4	1
Male	6	9	7	8	6	5	7	10	3
Diagnosis									
Leukemia	6	9	7	5	5	7	9	19	10
Solid tumor	2	1	2	2	2	2	1	1	0
Brain tumor	1	0	0	0	0	1	0	0	0
Other	1	0	1	1	3	0	0	0	0
Inpatient	5	6	2	4	4	1	2	6	3
Active treatment	9	10	8	8	9	8	9	16	9
Months since diagnosis									
0–6	9	5	2	4	4	3	2	7	4
> 6 to 12	0	1	1	1	1	2	1	2	3
> 12	1	4	7	5	5	5	7	11	3
Relapse	1	2	1	1	1	1	0	3	2
Hematopoietic Stem cell Transplant	1	1	2	1	1	2	0	2	0
Understood Second Language									
Hindi	1	2	0	0	0	2	1	1	1
Mandarin	2	1	0	1	0	0	2	0	0
Farsi	2	0	0	0	0	0	1	0	0
Other	1	0	0	0	2	2	0	3	4

^a^Number of patients

### Establishing questionnaire structure (cohorts 1–3)

Table 2 summarizes the results of the first phase to establish questionnaire structure. For the first cohort of 10 children, only 4/10 (40%) understood the time frame of yesterday, with many children describing events that happened more distant than yesterday. Thus, only today was tested in cohorts 2 and 3; this recall period was understood by 19/20 respondents.

**Table 2:** Establishing Questionnaire Structure – Proportion of Respondents Understanding Each Element (*N* = 30)^b^

	Cohort 1 (*n* = 10)				Cohort 2 (*n* = 10)				Cohort 3 (*n* = 10)			
	4 Year Olds (*n* = 3)	5 Year Olds (*n* = 3)	6 Year Olds (*n* = 4)	7 Year Olds (*n* = 0)	4 Year Olds (*n* = 5)	5 Year Olds (*n* = 1)	6 Year Olds (*n* = 3)	7 Year Olds (*n* = 1)	4 Year Olds (*n* = 5)	5 Year Olds (*n* = 1)	6 Year Olds (*n* = 3)	7 Year Olds (*n* = 1)
Recall Period^a^	1/3	2/3	1/4	–	4/5	1/1	3/3	1/1	5/5	1/1	3/3	1/1
Bother	0/3	1/3	2/4	–	4/5	1/1	3/3	1/1	4/5	1/1	3/3	1/1
3 Point Response Option Using Wong-Baker FACES Scale	1/3	3/3	4/4	–	5/5	1/1	1/3	1/1	5/5	1/1	3/3	1/1

^a^Recall period was “yesterday or today” for cohort 1 and “today” for cohorts 2 and 3

^b^Numerator is the number of patients who understood each element and denominator is the number of patients evaluated for understanding

In the initial 10 children, bother was asked with the time frame of “yesterday or today”. While only 3/10 of the initial cohort understood bother when asked with this recall period, we were uncertain whether lack of understanding was related to the recall period or the concept of bother itself. In cohorts 2 and 3, bother was asked with the recall period of today and was understood by 18/20 of these respondents.

The dichotomous response scale and the three 3-point Likert scales were tested in the first two cohorts among 20 respondents. The dichotomous response scale of yes/no was understood by all 20 (100%) respondents. In the testing of the three different 3-point Likert scales, 16/20 (80%) understood the Wong-Baker FACES, 14/20 (70%) understood FPS-R, and 13/20 (65%) understood the Pieces of Hurt scales. Given this data and as 12/20 (60%) preferred the Wong-Baker FACES scale, we used this scale in the remaining respondents and phases. Table 2 reflects understanding of this Likert scale.

In the last 10 respondents participating in this phase (cohort 3), all 10 (100%) understood the recall period of today, 9/10 (90%) understood the concept of bother and all 10 (100%) understood the 3-point Likert response scale using the Wong-Baker FACES.

### Understanding individual symptoms (cohorts 4–7)

Results of understanding of each mini-SSPedi item are illustrated in Table 3. Among the 40 respondents, 3 (7.5%) read all symptoms by themselves, 5 (12.5%) asked for some help with reading, and 32 (80%) asked for every symptom to be read aloud. Understanding in cohorts 4 and 5 were poor for five symptoms. Based upon responses, modifications starting in cohort 6 included changes to the symptom wording or enhancements to the synonym list. Among the 20 respondents in cohorts 4 and 5, only 10 (50%) understood “feeling disappointed or sad”, with five incorrectly understanding disappointed, believing it meant mad. Thus, this item was changed to “feeling sad” starting in cohort 6. Only 7 (35%) children in cohorts 4 and 5 understood “problems with thinking or remembering things” and thus, this item was changed to “forgetting things” based upon cognitive probing results. Similarly, “changes in how your face or body look” was not understood by any child and was modified to “changes in how you look” based upon qualitative feedback. As “tingly or numb hands or feet” was understood by only 9 (45%) children, it was changed to “hands or feet falling asleep or tingling”. Finally, only 6 (30%) children understood “changes in taste” and consequently, this item was changed to “food tastes different”.

**Table 3:** Understanding Individual Symptoms - Proportion of Respondents Understanding Each Symptom (*N* = 40)^b^

	Cohort 4 (*n* = 10)				Cohort 5 (*n* = 10				Cohort 6 (n = 10)				Cohort 7 (*n* = 10)			
	4 Year Olds (*n* = 6)	5 Year Olds (*n* = 3)	6 Year Olds (*n* = 1)	7 Year Olds (*n* = 0)	4 Year Olds (*n* = 1)	5 Year Olds (*n* = 6)	6 Year Olds (*n* = 3)	7 Year Olds (*n* = 0)	4 Year Olds (*n* = 1)	5 Year Olds (*n* = 5)	6 Year Olds (*n* = 1)	7 Year Olds (*n* = 3)	4 Year Olds (*n* = 2)	5 Year Olds (*n* = 5)	6 Year Olds (*n* = 0)	7 Year Olds (*n* = 3)
Feeling disappointed or sad^a^	1/6	1/3	0/1	–	0/1	5//6	3/3	–	1/1	5/5	1/1	3/3	2/2	5/5	–	3/3
Feeling scared or worried	6/6	3/3	1/1	–	1/1	6/6	3/3	–	1/1	5/5	1/1	3/3	2/2	5/5	–	3/3
Feeling cranky or angry	6/6	3/3	1/1	–	1/1	6/6	3/3	–	0/1	5/5	1/1	3/3	2/2	5/5	–	3/3
Problems thinking or remembering things^a^	2/6	1/3	1/1	–	0/1	1/6	2/3	–	1/1	5/5	1/1	3/3	2/2	5/5	–	3/3
Changes in how your body or face look^a^	0/6	0/3	0/1	–	0/1	0/6	0/3	–	0/1	5/5	1/1	3/3	2/2	5/5	–	3/3
Feeling tired	6/6	3/3	1/1	–	1/1	6/6	3/3	–	1/1	5/5	1/1	3/3	2/2	5/5	–	3/3
Mouth sores	5/6	2/3	1/1	–	0/1	5/6	2/3	–	0/1	1/5	1/1	0/3	2/2	5/5	–	3/3
Headache	5/6	3/3	1/1	–	1/1	5/6	3/3	–	0/1	5/5	1/1	3/3	2/2	5/5	–	3/3
Hurt or pain (other than headache)	6/6	3/3	1/1	–	0/1	5/6	2/3	–	1/1	5/5	1/1	3/3	2/2	5/5	–	3/3
Tingly or numb hands or feet^a^	1/6	2/3	1/1	–	0/1	3/6	2/3	–	0/1	1/5	1/1	3/3	1/2	4/5	–	3/3
Throwing up or feeling like you might throw up	6/6	3/3	1/1	–	1/1	6/6	3/3	–	1/1	4/5	1/1	3/3	2/2	5/5	–	3/3
Feeling more or less hungry than you usually do	6/6	3/3	1/1	–	0/1	6/6	3/3	–	1/1	5/5	1/1	3/3	2/2	5/5	–	3/3
Changes in taste^a^	0/6	1/3	0/1	–	0/1	2/6	3/3		0/1	5/5	1/1	3/3	2/2	5/5	–	3/3
Constipation (hard to poop)	5/6	3/3	1/1	–	1/1	5/6	2/3		0/1	3/5	1/1	1/3	2/2	5/5	–	3/3
Diarrhea (watery, runny poop)	5/6	3/3	1/1	–	0/1	4/6	3/3		0/1	4/5	1/1	3/3	2/2	5/5	–	3/3

^a^These items were modified starting in cohort 6 – see Table 4 and text

^b^Numerator is the number of patients who understood each element and denominator is the number of patients evaluated for understanding

In the final 10 respondents in this phase (cohort 7), 10 (100%) understood 14 symptoms with the assistance of the synonym list and 8 (80%) understood the one remaining symptom (“hands or feet falling asleep or tingly”).

### Understanding mini-SSPedi (cohorts 8 and 9)

In cohorts 8 and 9, all 30 children (100%) requested mini-SSPedi to be read aloud. Table 4 illustrates understanding of each mini-SSPedi item incorporating the modifications from the previous phases and iterative improvements to the synonym list. In cohort 9, all 10 children understood all 15 mini-SSPedi items.

**Table 4:** Understanding of Mini-SSPedi- Proportion of Respondents Understanding Each Symptom (*N* = 30)^a^

	Cohort 8 (*n* = 20)				Cohort 9 (*n* = 10)			
	4 Year Olds (*n* = 7)	5 Year Olds (*n* = 5)	6 Year Olds (*n* = 4)	7 Year Olds (*n* = 4)	4 Year Olds (*n* = 2)	5 Year Olds (*n* = 4)	6 Year Olds (*n* = 3)	7 Year Olds (*n* = 1)
Feeling sad^a^	7/7	5/5	4/4	4/4	2/2	4/4	3/3	1/1
Feeling scared or worried	7/7	5/5	4/4	4/4	2/2	4/4	3/3	1/1
Feeling cranky or angry	7/7	5/5	4/4	4/4	2/2	4/4	3/3	1/1
Forgetting things^a^	7/7	5/5	4/4	4/4	2/2	4/4	3/3	1/1
Changes in how you look^a^	7/7	5/5	4/4	4/4	2/2	4/4	3/3	1/1
Feeling tired	7/7	5/5	4/4	4/4	2/2	4/4	3/3	1/1
Mouth sores	5/7	3/5	3/4	3/4	2/2	4/4	3/3	1/1
Headache	7/7	5/5	4/4	4/4	2/2	4/4	3/3	1/1
Hurt or pain (other than headache)	7/7	5/5	4/4	4/4	2/2	4/4	3/3	1/1
Hands or feet falling asleep or tingling^a^	7/7	5/5	4/4	4/4	2/2	4/4	3/3	1/1
Throwing up or feeling like you might throw up	7/7	5/5	4/4	4/4	2/2	4/4	3/3	1/1
Feeling more or less hungry than you usually do	7/7	5/5	4/4	4/4	2/2	4/4	3/3	1/1
Food tastes different^a^	7/7	4/5	4/4	4/4	2/2	4/4	3/3	1/1
Constipation (hard to poop)	7/7	4/5	4/4	4/4	2/2	4/4	3/3	1/1
Diarrhea (watery, runny poop)	7/7	5/5	4/4	4/4	2/2	4/4	3/3	1/1

^a^These items were modified starting in cohort 6 – see Table 3

^b^Numerator is the number of patients who understood each element and denominator is the number of patients evaluated for understanding

When questioned about the length of mini-SSPedi (cohort 9), 7 (70%) children stated the instrument was “about right” and 3 (30%) stated the instrument was too short. When evaluating ease of completion, no child (0%) stated mini-SSPedi was hard to complete. When evaluated by parents, all judged mini-SSPedi was easy or very easy for the child to complete.

**Fig. 1:**
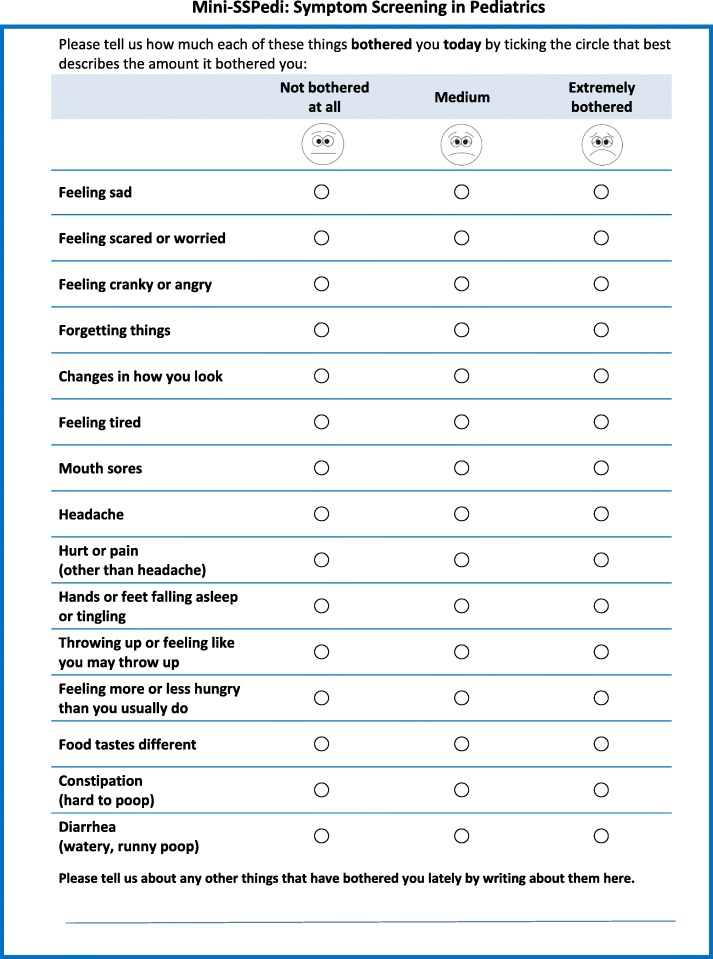
Mini-SSPedi: Symptom Screening in Pediatrics

Mini-SSPedi was finalized after cohort 9; Fig. 1 illustrates the final version of mini-SSPedi.

## Discussion

Using an iterative approach with cognitive interviewing, we successfully developed a paper version of mini-SSPedi that is understandable and easy to complete by English-speaking children with cancer and pediatric HSCT recipients who are 4 to 7 years of age. This research is important as developing a symptom screening tool for children in this younger age group is necessary to improve symptom control in this population.

Building upon procedures developed by others [7, 9, 10, 12], we learned important methodological lessons during the study and used unique approaches for this younger age group. First, we found that creation and evaluation of the instrument required a more phased approach compared to the development of SSPedi for older children and adolescents. Second, we found that when the instrument is read aloud, optimal understanding of mini-SSPedi required a specific approach to administration and pointing to the faces response scale with each symptom. Finally, this study emphasizes that the majority of children in this age group will require the instrument to be read aloud during future research and clinical utilization. Therefore, electronic mini-SSPedi should be created such that reading the instrument aloud is the default setting.

A unique aspect of this study is the large number of children required to produce a satisfactory version of mini-SSPedi. This large number was required as we felt we had to establish the appropriate structure of the instrument including the recall period and response scale before testing symptom understanding. We then needed to evaluate understanding of each symptom prior to testing of the instrument in its entirety. We believe this step-wise approach is ideal toward creating an understandable instrument for this age range.

The strengths of this study were the rigorous and iterative approach implemented in the development of mini-SSPedi. Second, we relied upon a multidisciplinary group of healthcare experts, including a patient advocate and a clinical psychologist, to constitute the Review Panel. The third strength is symmetry with SSPedi, which should allow combined analyses with younger and older children. However, our study has several limitations including its conduct at a single center and availability of mini-SSPedi only in English. Future efforts will include testing psychometric properties in a multi-center approach and translation to other languages.

## Conclusion

We developed a symptom screening tool for children with cancer and pediatric HSCT recipients between 4 and 7 years of age that is understandable and easy to complete. Future work will evaluate the psychometric properties of mini-SSPedi and develop an electronic version of the instrument.

## Additional file

Additional file 1: 3-point Likert response scale - pictorial options. (DOCX 77 kb)
