# Supplementary material for: Development of mini-SSPedi for children 4–7 years of age receiving cancer treatments
Source: BMC Cancer. 2019 Jan 8;19:32. doi: 10.1186/s12885-018-5210-z (PMC6325666; doi:10.1186/s12885-018-5210-z)
Supplement: Supplementary file 1 — 3-point Likert response scale - pictorial options. (DOCX 77 kb) [file 12885_2018_5210_MOESM1_ESM.docx]

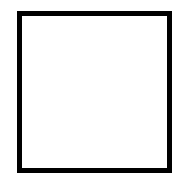

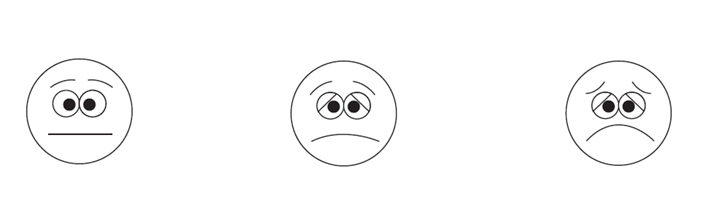

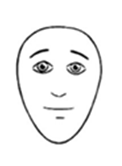

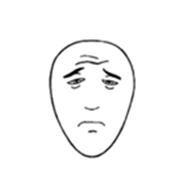

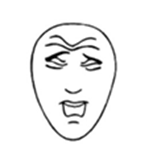

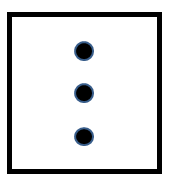

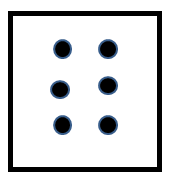


**Additional file 1: 3-point Likert response scale - pictorial options***

i) Wong-Baker FACES Scale

ii) Faces Pain Scale-Revised

iii) Pieces of Hurt

* Each scale was presented on a separate page, without wording
